# Long non-coding RNA PCED1B-AS1 promotes the proliferation of colorectal adenocarcinoma through regulating the miR-633/HOXA9 axis

**DOI:** 10.1080/21655979.2022.2037225

**Published:** 2022-02-17

**Authors:** Jianfeng Liu, Jun Qian, Qi Mo, Liming Tang, Qiang Xu

**Affiliations:** Department of Gastrointestinal Surgery, Changzhou No. 2 People’s Hospital of Nanjing Medical University, Changzhou City, Jiangsu Province, PR, China

**Keywords:** Colorectal adenocarcinoma, PCED1B-AS1, miR-633, HOXA9, Tumorigenicity

## Abstract

Long non-coding RNA (lncRNA) PCED1B-AS1 was shown to play essential roles in human cancers, while its function in colorectal adenocarcinoma remains unclear. This study was carried out to investigate the function of PCED1B-AS1 in regulating the microRNA(miR)-633/HOXA9 axis in colorectal adenocarcinoma. The expression of PCED1B-AS1, miR-633 and HOXA9 was measured by quantitative real-time PCR (qRT-PCR) or Western blot analysis. Cell behaviors of colorectal adenocarcinoma cell lines were assessed by CCK-8, EdU, Transwell and flow cytometry assays. The interaction among PCED1B-AS1, miR-633 and HOXA9 was determined by luciferase reporter and RIP assays. Rescue experiments were performed to determine the regulatory axis in colorectal adenocarcinoma. Moreover, an animal model was established to verify the role of PCED1B-AS1. We found that PCED1B-AS1 was upregulated and miR-633 was downregulated in colorectal adenocarcinoma tissues and corresponding cell lines. Knockdown of PCED1B-AS1 inhibited cell proliferation and promoted apoptosis, while miR-633 inhibitor elevated proliferation and reduced apoptosis of cancer cell lines. In addition, overexpression of HOXA9 obviously attenuated the protective role of knockdown of PCED1B-AS1 or miR-633 mimics in colorectal adenocarcinoma progression. PCED1B-AS1 could negatively regulate the expression of HOXA9 by sponging miR-633. The *in vivo* experiments confirmed the role of PCED1B-AS1 and miR-633 in colorectal adenocarcinoma, as well as the regulatory relationship of this axis. Our results demonstrated that knockdown of PCED1B-AS1 inhibited the progression of colorectal adenocarcinoma by regulating the miR-633/HOXA9 axis.

## Graphical abstract

**Figure:**
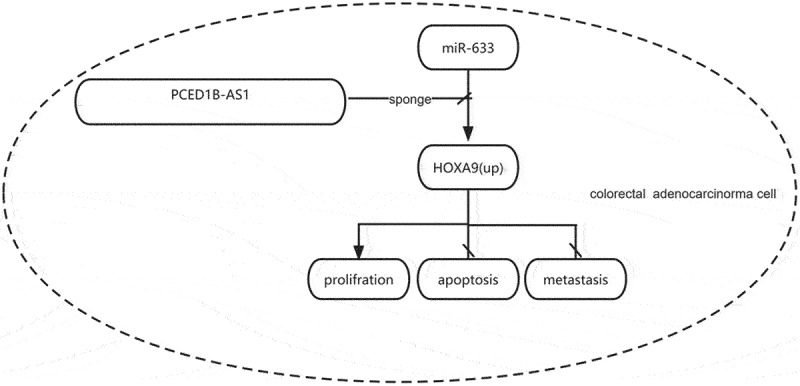

## Introduction

Colorectal cancer is becoming the third most common cancer and has second higher mortality in the world [1]. Approximately 50% of the colorectal adenocarcinoma patients will develop liver metastasis and 20% of that are synchronic [2]. The current therapeutic strategies for this disease are surgery, chemotherapy and radiotherapy [3]. Although extensive efforts have been made, the prognosis of this disease is still poor [4]. Therefore, a better understanding of the potential mechanisms involved in colorectal adenocarcinoma progression is urgent.

As the novel type of regulators, long non-coding RNAs (lncRNAs) have been demonstrated to play important roles in the regulation of cancer progression [5]. LncRNAs have been identified to participate in cancer development by acting as the sponges for microRNAs (miRNAs) [6]. There are also other tumorigenesis mechanisms for lncRNAs. For example, lncRNAs may serve as activator to promote transcription, as suppressor to repress transcription, as epigenetic regulators, or as scaffolds to form ribonucleoprotein complex [7]. The involvement and functions of lncRNAs have also been investigated in colorectal adenocarcinoma. For example, MEG3 inhibits cell proliferation and promotes the apoptosis of colorectal adenocarcinoma cells by up-regulating TGF-beta1 [8]. Downregulation of LINC01638 suppresses cell proliferation of colorectal adenocarcinoma cells by interacting with RUNX2 [9]. DSCAM-AS1 promotes the invasion and migration of colorectal adenocarcinoma cells by downregulating miR-216b [10]. PC‑esterase domain containing 1B‑antisense RNA 1 (PCED1B‑AS1) is a recently identified lncRNA that was reported to participate in the progression of several human cancers such as pancreatic ductal adenocarcinoma [11], clear cell renal cell carcinoma [12], hepatocellular carcinoma [13], and glioma [14]. However, the role of PCED1B‑AS1 in colorectal adenocarcinoma remains unclear.

In the present study, we found that PCED1B-AS1 was upregulated and miR‐633 was downregulated in colorectal adenocarcinoma tissues and corresponding cell lines. Besides, the expression of PCED1B-AS1 and miR‐633 was negatively correlated with each other. These results indicate the potential regulatory relationship between PCED1B-AS1 and miR‐633. In this study, we aimed to investigate the role of PCED1B-AS1 in colorectal adenocarcinoma tissues and to identify the potential relationship among PCED1B-AS1, miR-633 and HOXA9 in colorectal adenocarcinoma.

## Materials and methods

### Tissue samples

A total of 40 pairs of colorectal adenocarcinoma tissues and matched adjacent normal tissues were collected from 40 cancer patients through surgical excision at Changzhou No. 2 People’s Hospital of Nanjing Medical University between 2016 to 2020. For the selection patients, inclusion criteria were as follows: 1) no adjunctive treatment prior to surgery; 2) pathologically diagnosed as colorectal adenocarcinoma by two senior pathologists; 3) surgical excision performed between 2016 and 2020. The exclusion criteria were as follows: patients complicated with other malignant tumors, with incomplete clinical or prognostic data. The paired adjacent normal tissues (5 cm from colorectal adenocarcinoma tissues) and colorectal adenocarcinoma tissues were obtained from 40 patients. All samples were frozen in liquid nitrogen and stored at −80°C before use. The clinicopathological features of 40 cancer patients were listed in Supplementary Table 1. This study was approved by the Ethics Committee of the aforementioned hospital and conducted in compliance with the guidelines of the World Medical Association Declaration of Helsinki. Written informed consent was obtained from all enrolled patients.

### Cell culture

The normal colonic epithelial cell line NCM460 and colorectal adenocarcinoma cell lines LS-174 T, SW620, HCT-8, LoVo were obtained from ATCC (Manassas, VA, USA). Cells were cultured in DMEM medium (PH7.4, Gibco, New York, USA) containing 10% FBS (FBS, Sigma-Aldrich, MO, USA) in a 37°C incubator with an additional 5% CO_2,_ 100 U/ml penicillin and 100 µg/ml streptomycin (1% Pen-Strep, Invitrogen).

### Cell transfection

The short hairpin RNA for PCED1B-AS1 (sh-PCED1B-AS1), and negative control sh-NC, miR-633 mimics, miR-633 inhibitor and corresponding controls (miR-NC and inhibitor NC) were designed and synthesized by GenePharma (Shanghai, China). In addition, the overexpressing vector pcDNA3.1 carrying the full length of PCED1B-AS1 and HOXA9 were also constructed by GenePharma to generate pcDNA-PCED1B-AS1 and pcDNA-HOXA9. The empty vector pcDNA3.1 was used as the negative control. Cell transfection was conducted using Lipofectamine 3000 (Invitrogen). The sequences were as follows.. miR-633 mimics, 5′-CUAAUAGUAUCUACCACAAUAAA-3′; mimic-NC 5′-UUCUCCGAACGUGUCACGUTT-3′; miR-633 inhibitor, 5′-UUUAUUGUGGUAGAUACUAUUAG-3′; inhibitor-NC, 5′-CAGUACUUUUGUGUAGUACAA-3′. sh-PCED1B-AS1: 5′-ATTTGGAGCTGTTGTCCATTA-3′; and sh-NC: 5′-ATGGGCCGCTGGTGCCCAATT-3′.

## Quantitative real-time PCR (*qRT-PCR)*

Total RNAs of tissue samples and cells were extracted using Trizol reagent (Thermo Fishier Scientific). The cDNA was synthesized using the reverse transcription kit (Takara, Dalian, China). PCR reactions were performed on the 7500 real‑time PCR machine (Applied Biosystems). The primers of cDNA synthesis of miRNA and U6 were obtained from a RT primer pool with reverse transcriptase supplied in the kit. Relative expression was calculated using the 2^−ΔΔCt^ method [15] with GAPDH and U6 as the internal controls. The primers used were as follows: PCED1B-AS1 F: 5′-TCAAGCCAATCAGCTGACAC-3′, R: 5′-AAACAAATGCCCTGCTTGAC-3′; miR-633 F: 5′-CCGATACGATGAGAGAAACCCTGA-3′, R: 5’-GGACAGAGTTGACTTAAGGCTAGA-3’; HOXA9 F: 5’-CAACAAAGACCGAGCAAA-3’, R: 5’- CAACAAAGACCGAGCAAA-3′; GAPDH F: 5′-ACCCAGAAGACTGTGGATGG-3′, R: 5′-TTCAGCTCAGGGATGACCTT-3′; U6 F: 5′-CTCGCTTCGGCAGCACA-3′, R: 5′-AACGCTTCACGAATTTGCGT-3′.

### Western blot analysis

Total proteins were extracted using RIPA lysis buffer (Beyotime, China). Protein concentration was determined using the BCA method (abcam, USA). Next, 1 μg protein sample was separated by 12% SDS-PAGE and transferred onto PVDF membranes. After blocking, the membranes were incubated with primary antibodies against HOXA9 (1:1,000; Abcam, ab140631) and GAPDH (1:2,000; Abcam, ab8245). On the next day, the horseradish peroxidase-labeled IgG (1:500, Abcam) was added for incubation for another 2 h. The immunoblots were detected using the enhanced chemiluminescence kit (Thermo Fishier Scientific), and the relative expression levels of protein were calculated as the ratio of the gray value of the target band to GAPDH band using a Bio-Rad imaging system (Bio-Rad GelDoc XR+ image software).

### Cell proliferation

For cell viability, a total of 1,000 cells/100 μL per well were cultured in five replicate wells in a 96-well plate in medium containing 10% FBS. Cells were cultured for 24, 48, 72 and 96 h. Then 10 ul CCK8 reagent (included in the Cell Counting Kit-8, Solarbio) was added to each well and incubated for 2 h. The absorbance at 450 nm was detected using a microplate reader (BioTek, Winooski, VT, USA). For cell proliferation, the EdU Staining Proliferation Kit (Abcam) was used following the manufacturer’s instructions. The cells were observed under a fluorescence microscope (NIikon, ELCIPSE-CI) with the filter set as Ex/Em = 491/520 nm, and images were captured by CIM-2.

### Cell apoptosis assay

Cell apoptosis was assessed using the Annexin V-FITC/propidium Iodide (PI) Apoptosis Detection kit (Sigma-Aldrich). In brief, 5 × 10^4^ cells were incubated with 5 ul of Annexin V-FITC and PI solution for 12 min. The apoptotic rate was analyzed by FACS cytometer (BD Biosciences, FACS canto II, USA). Data were acquired with a BD Biosciences (BD) LSRFortessa equipped with 5 lasers (355-, 407-, 488-, 532-, and 633-nm wavelengths). The signal was detected by 22 PMT detectors using the DIVA 8 software. The model used the High Throughput Sampler system (BD) at a flow rate of 2.5 μl/second in a 96-well U-bottom tissue culture plate. Compensation controls were performed using single-color staining of compensation beads (BD) following the manufacturer’s recommendations. Analysis after acquisition was performed using the FlowJo version 10 software (Tree Star Inc).

### Transwell assay

The invasion and migration of cancer cells were detected by Transwell assay using Transwell membrane coating with or without Matrigel™ (BD Biosciences) as previously described [16]. The average number of invasive and migrated cells were captured and counted under a light microscopy (objective: 10x, magnification: 200x; Nikon, Tokyo, Japan) in five random fields.

### Luciferase reporter assay

The potential binding site between PCED1B-AS1, miR-633 and HOXA9 was predicted by Starbase and Targetscan [17,18]. The luciferase reporter vector carrying the wild type (WT) and mutant (MUT) PCED1B-AS1 or HOXA9 containing miR-633 binding site were constructed by GenePharma to generate Luc-PCED1B-AS1-WT/MUT and Luc-HOXA9-WT/MUT (map was shown in Supplement Data 1). The recombinant luciferase vectors were co-transfected with miR-633 or miR-NC into SW620 and LoVo cells by using Lipofectamine 3000. After 48 h, the relative luciferase activity was determined using the dual-luciferase reporter assay system (Promega) [15].

### RNA immunoprecipitation (RIP) assay

To identify the direct sponge relationship between PCED1B-AS1 and miR-633 *via* Ago2, Ago-RNA immunoprecipitation (RIP) assay was employed. RIP assay was conducted using the EZMagna RIP kit (Millipore). In brief, the cell lysate was incubated with magnetic beads conjugated with anti-Ago2 or control anti-IgG for 4 h. The enriched RNAs were released from the beads by proteolysis in 100 μL of Elution Buffer (ThermoFisher Scientific) and 4 U RNaseOUT at 42°C for 1 h, followed by 55°C for 1 h. Then RNA was used for qRT-PCR analysis.

### Animal model

For *in vivo* experiments, 5 × 10^6^ SW620 cells transfected with short hairpin-PCED1B-AS11 (Sh-PCED1B-AS1), short hairpin negative control (sh-NC), miR-633 inhibitor or co-transfected with Sh-PCED1B-AS1 and miR-633 inhibitor were separately subcutaneously inoculated into the left flank in the dorsal of the male nude BALB/c mice (n = 6/group). Tumor size was evaluated every one week for five weeks, and tumor volume was measured using the formula (length × width^2^)/2. Five weeks later, mice were sacrificed, and tumors were fetched and weighed. All animal experiments were approved by the Animal Ethics Committee of Changzhou No. 2 People’s Hospital of Nanjing Medical University.

### Immunochemistry assay

For the detection of cell proliferation *in vivo*, the immunochemistry assay was performed by incubating tumor tissues with anti-Ki67 antibody (1:200, Abcam, cat#ab15580) as previously described [19]. The images were captured using an Olympus BX 41 light microscope (magnification: 100x and 400x, objective:10x) with the visual mode analySIS 3.2 software.

### Statistics analysis

All data were presented as mean ± standard deviation (SD) using the SPSS 18.0 software. Difference between groups was determined by Student’s t test or one-way ANOVA. P < 0.05 was considered as the significant threshold.

## Results

### PCED1B-AS1 was upregulated and miR‐633 was downregulated in colorectal adenocarcinoma

**Figure 1.**
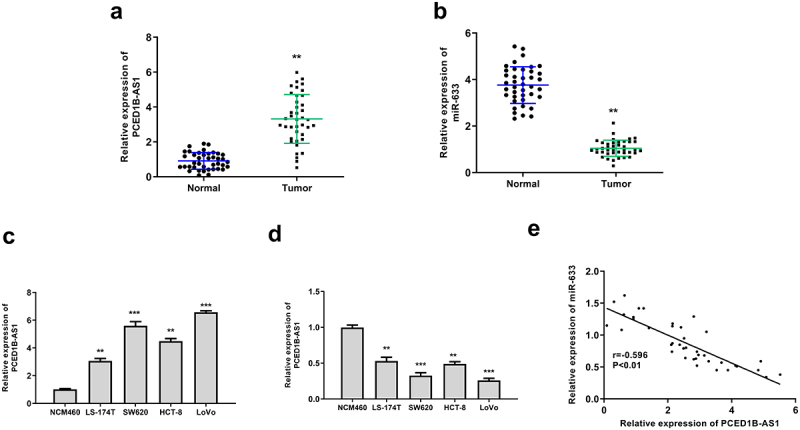
PCED1B-AS1 was upregulated and miR‐633 was downregulated in colorectal adenocarcinoma. (a and b) The mRNA levels of PCED1B-AS1 (a) and miR‐633 (b) in colorectal adenocarcinoma tissues were evaluated by qRT-PCR (n = 40). (c and d) The mRNA levels of PCED1B-AS1 (c) and miR‐633 (d) in colorectal adenocarcinoma cell lines were evaluated by qRT-PCR. (e) The relationship between the levels of PCED1B-AS1 and miR‐633 in colorectal adenocarcinoma tissues. * *p* < 0.05, ** *p* < 0.01.

To evaluate the correlation of PCED1B-AS1 and miR‐633 in colorectal adenocarcinoma, RT-qPCR was performed to evaluate their expression in cancer tissue and cell lines. The results showed that PCED1B-AS1 was upregulated (*p* < 0.01) and miR‐633 was downregulated (*p* < 0.01) in colorectal adenocarcinoma tissues (Figure 1a and b). In cancer cell lines, the expression patterns of PCED1B-AS1 and miR‐633 were consistent with that in tumor tissues (*p* < 0.01 or *p* < 0.001, Figure 1c and d). Due to the higher expression levels of PCED1B-AS1 and lower expression levels of miR-633 in SW620 and LoVo cells, these two cell lines were selected for the subsequent analyses. In addition, there was an obvious negative correlation between the expression levels of PCED1B-AS1 and miR‐633 in colorectal adenocarcinoma tissues (*p* < 0.01, Figure 1e). These data suggested that PCED1B-AS1 and miR‐633 play potential roles in colorectal adenocarcinoma.

### Knockdown of PCED1B-AS1 significantly inhibited the progression of colorectal adenocarcinoma cells

**Figure 2.**
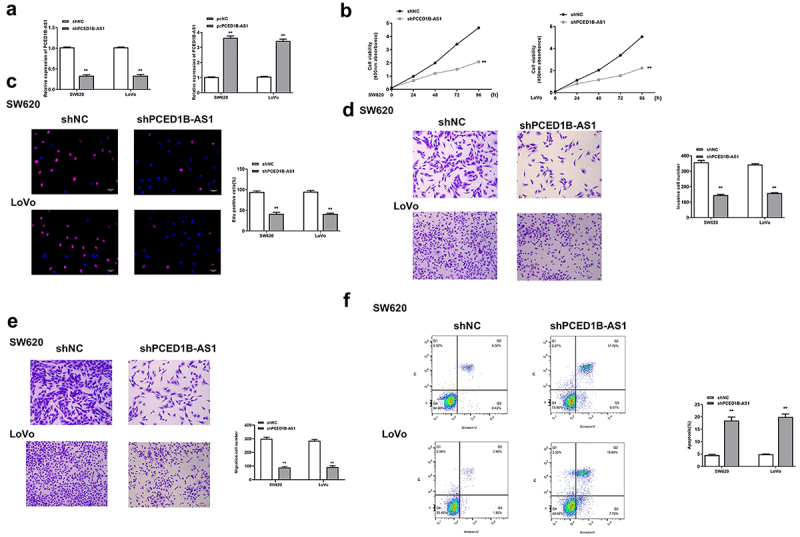
Knockdown of PCED1B-AS1 significantly inhibited the progression of colorectal adenocarcinoma cells. SW620 and LoVo cells were transfected with sh-PCED1B-AS1 and pc-PCED1B-AS1 (overexpressing vector). (a) The mRNA levels of PCED1B-AS1 was detected by qRT-PCR. (b) CCK-8 assay. (c) EdU staining assay. Scale bar = 100 μm. (d and e) The invasion and migration of cells was evaluated by Transwell assay. Scale bar = 100 μm. (f) results of flow cytometry assay, FS/SS was used to remove debris, FS-H/fs-a was used to remove adherent cells, and the cells were analyzed in Flt1/FLT3 channel. ** *p* < 0.01.

To determine the role of PCED1B-AS1 in colorectal adenocarcinoma cells, CCK8 assay, transwell assay and fllow cytometry were performed to detect the effect of overexpression or silencing of PCED1B-AS1 on the cells. sh-PCED1B-AS1 and pc-PCED1B-AS1 (overexpressing vector) were transfected into SW620 and Lovo cells, and the transfection efficiency was determined by qRT-PCR (all *p* < 0.01, Figure 2a). The results showed that knockdown of PCED1B-AS1 significantly reduced the proliferation (all *p* < 0.01, Figure 2b and c), invasion and migration (all *p* < 0.01, Figure 2d and e), while promoted the apoptosis of SW620 and Lovo cells (both *p* < 0.01, Figure 2f). These results demonstrated that knockdown of PCED1B-AS1 significantly inhibited the progression of colorectal adenocarcinoma.

### Overexpression of miR-633 inhibited the progression of colorectal adenocarcinoma cells

**Figure 3.**
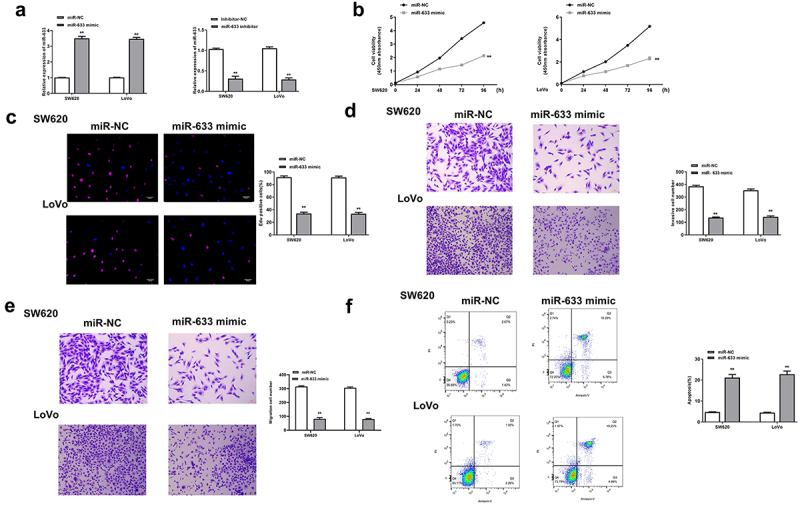
Overexpression of miR-633 inhibited the progression of colorectal adenocarcinoma cells. SW620 and LoVo cells were transfected with miR-633 mimics and inhibitor. (a) The mRNA levels of miR-633 were detected by qRT-PCR. (b) CCK-8 assay. (c) EdU staining assay. Scale bar = 100 μm. (d and e) The invasion and migration of cells was evaluated by Transwell assay. Scale bar = 100 μm. (f) flow cytometry assay. FS/SS was used to remove debris, FS-H/fs-a was used to remove adherent cells, and the cells were analyzed in Flt1/FLT3 channel. ** *p* < 0.01.

Next, the function of miR-633 was investigated in colorectal adenocarcinoma cells. The miR-633 mimics and inhibitor were transfected into SW620 and Lovo cells, and the transfection efficiency was determined by qRT-PCR (all *p* < 0.01, Figure 3a). Similarly, miR-633 mimics significantly reduced the proliferation (*p* < 0.01, Figure 3b and c), invasion and migration (all *p* < 0.01, Figure 3d and e), while promoted the apoptosis of SW620 and Lovo cells (both *p* < 0.01, Figure 3f). These results demonstrated that the effect of miR-633 was similar to that of knockdown of PCED1B-AS1.

### PCED1B-AS1 negatively regulated miR-633

**Figure 4.**
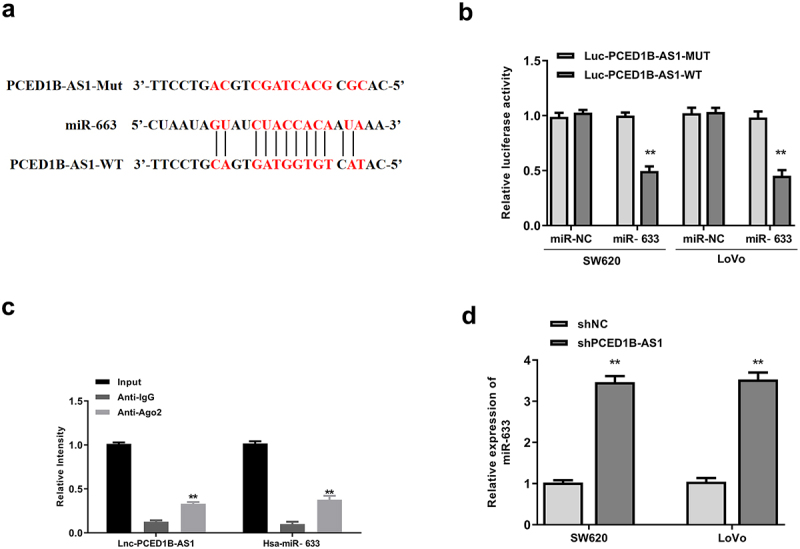
PCED1B-AS1 acted as the sponge of miR-633. (a) The putative binding site between PCED1B-AS1 and miR‐633 by Starbase. (b) Luciferase reporter assay. (c) RIP assay. (d) The mRNA level of miR‐633 in SW620 and LoVo cells transfected with sh-PCED1B-AS1 was measured by qRT-PCR. ** *p* < 0.01.

Based on the negative correlation of PCED1B-AS1 and miR-633 in colorectal adenocarcinoma cells, the possible sponge relationship between them was then investigated. Starbase was used to predict the potential targets of PCED1B-AS1. It showed putative binding sites between PCED1B-AS1 and miR-633 (Figure 4a). Luciferase reporter assay showed that miR-633 mimics significantly reduced the relative luciferase activity of Luc-PCED1B-AS1-WT (both *p* < 0.01), and had no impact on Luc-PCED1B-AS1-MUT in the two cell lines (Figure 4b). To identify the directly sponge relationship between PCED1B-AS1 and miR-633, Ago-RNA immunoprecipitation (RIP) assay was performed. RIP assay results showed that both PCED1B-AS1 and miR-633 were enriched in immune-complex of anti-Ago2 group compared with that in anti-IgG group (all *p* < 0.01, Figure 4c). Moreover, knockdown of PCED1B-AS1 significantly elevated the expression levels of miR-633 in two cell lines compared with that of sh-NC (both *p* < 0.01, Figure 4d). These results suggested that PCED1B-AS1 could bind to miR-633 and negatively regulate its expression.

### MiR-633 inhibitor partially eliminated the inhibitory effect of sh-PCED1B-AS1 on colorectal adenocarcinoma progression

**Figure 5.**
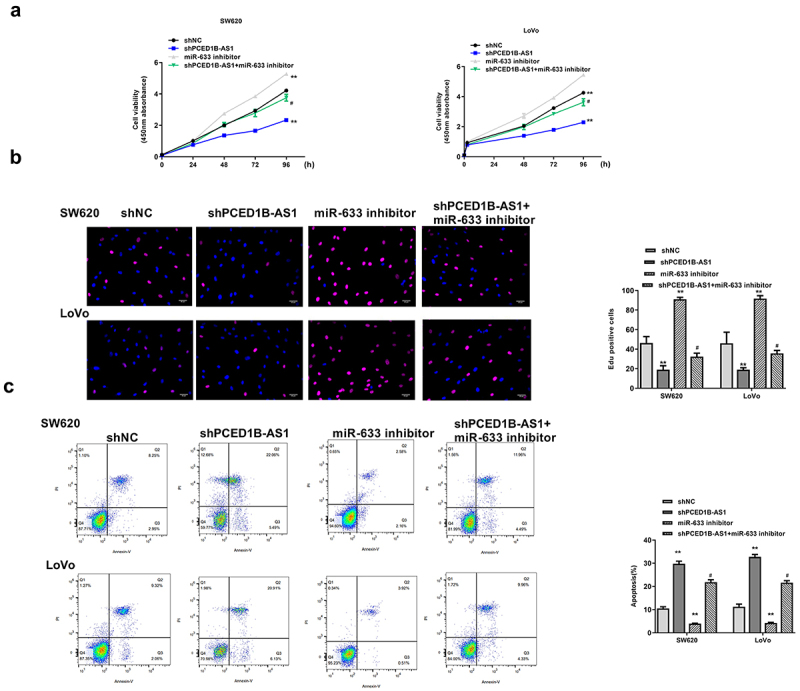
MiR-633 mediate the function of PCED1B-AS1. SW620 and Lovo cells were transfected with sh-PCED1B-AS1, miR-633 inhibitor, or co-transfected with sh-PCED1B-AS1 and miR-633 inhibitor. (a) CCK-8 assay. (b) EdU staining assay. Scale bar = 100 μm. (c) flow cytometry assay. FS/SS was used to remove debris, FS-H/fs-a was used to remove adherent cells, and the cells were analyzed in Flt1/FLT3 channel ** *p* < 0.01 vs sh-NC and # *p* < 0.05 vs sh-PCED1B-AS1.

Next, whether miR-633 could rescue the impact of sh-PCED1B-AS1 on colorectal adenocarcinoma progression was investigated. SW620 and Lovo cells were transfected with sh-PCED1B-AS1, miR-633 inhibitor, or co-transfected with sh-PCED1B-AS1 and miR-633 inhibitor. We found that miR-633 inhibitor exacerbated the progression of colorectal adenocarcinoma, which was indicated by elevated cell proliferation (all *p* < 0.01, Figure 5a and b) and reduced apoptosis of SW620 and LoVo cells (both *p* < 0.01, Figure 5c), while co-transfection with sh-PCED1B-AS1 and miR-633 inhibitor partially eliminated the inhibitory impact of sh-PCED1B-AS1 on colorectal adenocarcinoma progression (*p* < 0.05, Figure 5a-c). These results demonstrated that miR-633 mediated the function of PCED1B-AS1.

### HOXA9 was a target of miR-633

**Figure 6.**
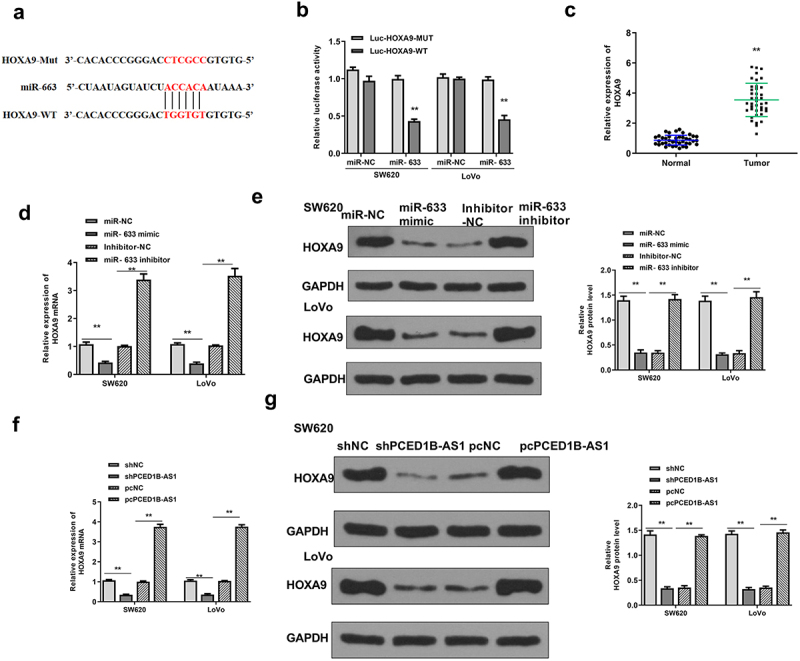
HOXA9 was a target of miR-633. (a) The putative binding site between miR‐633 and HOXA9 by Targetscan. (b) Luciferase reporter assay. (c) The mRNA level of HOXA9 in colorectal adenocarcinoma tissues were evaluated by qRT-PCR (n = 40). (d and e) SW620 and Lovo cells were transfected with miR-633 mimics or inhibitor. The expression of HOXA9 was detected by qRT-PCR (d) and Western blot (e). (f and g) SW620 and Lovo cells were transfected with sh-PCED1B-AS1 or pc-PCED1B-AS1 (overexpressing vector). The expression of HOXA9 was detected by qRT-PCR (f) and Western blot (g). ** *p* < 0.01.

Next, the downstream target gene of miR-633 was explored. Targetscan was used to search for potential target genes of miR‐633, and the prediction results showed that miR-633 might bind to the 3′-UTR of miR‐633 (Figure 6a). Luciferase reporter assay revealed that miR-633 mimics significantly reduced the relative luciferase activity of Luc-HOXA9-WT (both *p* < 0.01), but had no impact on Luc-HOXA9-MUT in the two cell lines (Figure 6b). The expression of HOXA9 was significantly upregulated in colorectal adenocarcinoma tissues (*p* < 0.01, Figure 6c). Meanwhile, overexpression of miR-633 reduced the expression levels of HOXA9, and downregulation of miR-633 elevated the expression levels of HOXA9 (all *p* < 0.01, Figure 6d and e). Moreover, knockdown of PCED1B-AS1 downregulated HOXA9 and overexpression of PCED1B-AS1 upregulated its expression (all *p* < 0.01, Figure 6f and g). These results suggested that HOXA9 was a target of miR-633.

### Overexpression of HOXA9 partially eliminated the inhibitory effect of miR-633 mimics in colorectal adenocarcinoma progression

**Figure 7.**
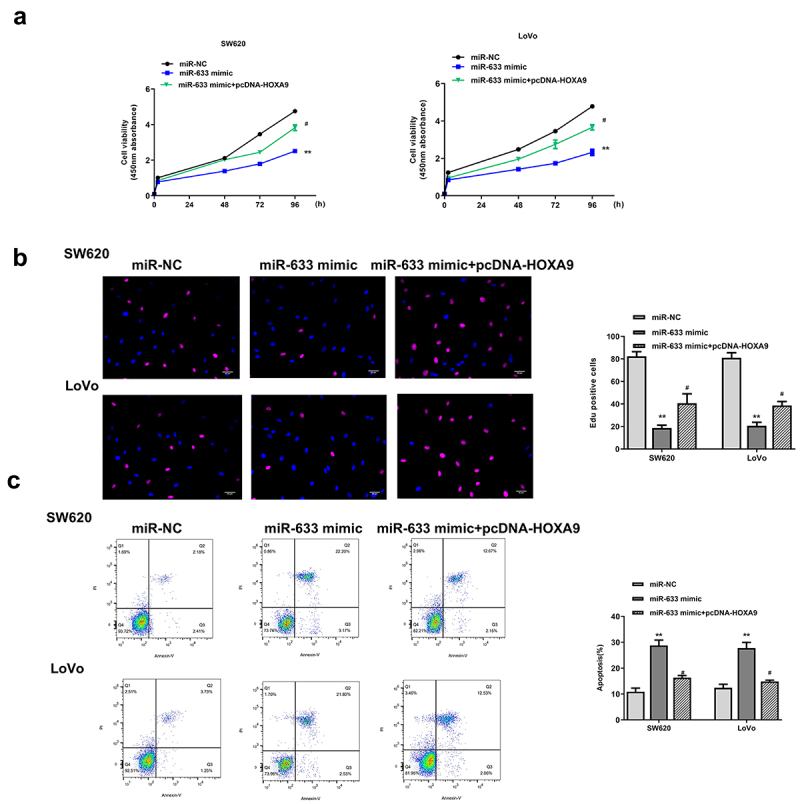
Overexpression of HOXA9 partially eliminated the inhibitory impacts of miR-633 mimics in colorectal adenocarcinoma progression. SW620 and Lovo cells were transfected with miR-633 mimics, miR-NC, or co-transfected with miR-633 mimics and pcDNA-HOXA9. (a) CCK-8 assay. (b) EdU staining assay. Scale bar = 100 μm. (c) flow cytometry assay. FS/SS was used to remove debris, FS-H/fs-a was used to remove adherent cells, and the cells were analyzed in Flt1/FLT3 channel. ** *p* < 0.01 vs miR-NC and # *p* < 0.05 vs miR-633 mimics.

To explore whether miR‐633 regulated colorectal adenocarcinoma progression through HOXA9, trans- or co-transfection experiment of miR-633 mimics and pcDNA-HOXA9 was performed in SW620 and Lovo cells. CCK-8 assay, EdU staining assay and flow cytometry assay were then performed to investigate the possible rescue effect of the HOXA9 on the effect of miR-633. SW620 and Lovo cells were transfected with miR-633 mimics, miR-NC, or co-transfected with miR-633 mimics and pcDNA-HOXA9. We found that overexpression of miR-633 obviously reduced cell proliferation and promoted apoptosis (all *p* < 0.01), while co-transfection of miR-633 mimics and pcDNA-HOXA9 significantly attenuated the inhibitory effect of miR-633 mimics in colorectal adenocarcinoma progression (all *p* < 0.05, Figure 7a-c). These data demonstrated that miR-633 affectsed colorectal adenocarcinoma progression by regulating HOXA9.

### Knockdown of PCED1B-AS1 inhibited the tumorigenicity of colorectal adenocarcinoma in vivo

**Figure 8.**
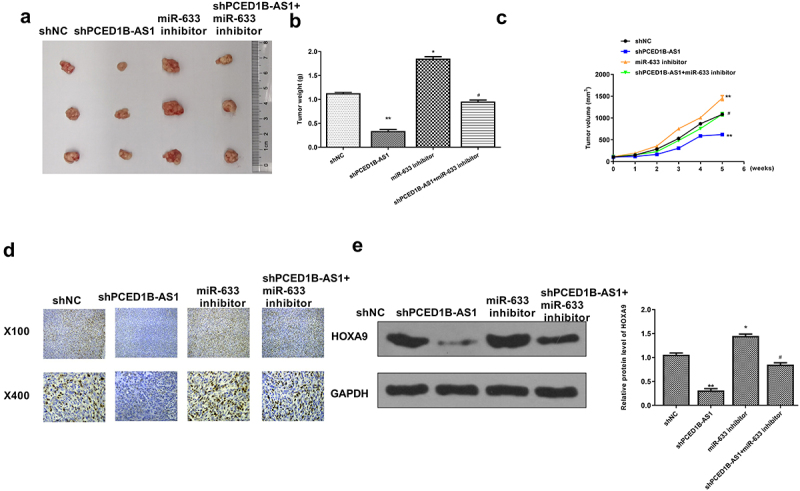
Knockdown of PCED1B-AS1 inhibited the tumorigenicity of colorectal adenocarcinoma *in vivo*. (a) The representative images of subcutaneous tumors. (b) Tumor weight. (c) Tumor volume. (d) Ki67 immunochemistry assay. (f) The protein level of HOXA9 in tumors was evaluated by Western blot. * *p* < 0.05, ** *p* < 0.01 vs sh-NC and # *p* < 0.05 vs sh-PCED1B-AS1.

Finally, an *in vivo* animal model was constructed to confirm the role of the PCED1B-AS1/miR-633-HOXA9 axis in the tumor bearing mice. As shown in Figure 8a-c, knockdown of PCED1B-AS1 obviously inhibited the tumor growth, and miR-633 promoted tumor growth, while co-transfection of sh-PCED1B-AS1 and miR-633 inhibitor attenuated the protective role of sh-PCED1B-AS1 in tumor progression (*p* < 0.05 and *p* < 0.01, Figure 8a-c). Ki-67 immunochemistry staining showed that sh-PCED1B-AS1 reduced cell proliferation *in vivo*, while co-transfection of sh-PCED1B-AS1 with miR‐633 inhibitor reversed the effect of sh-PCED1B-AS1 on cell proliferation (Figure 8d). In addition, knockdown of PCED1B-AS1 reduced the expression levels of HOXA9 (*p* < 0.01), and miR-633 elevated the expression levels of HOXA9 (*p* < 0.05), while co-transfection attenuated the effect of sh-PCED1B-AS1 on the expression of HOXA9 (*p* < 0.05, Figure 8e). These results confirmed the regulation of the PCED1B-AS1/miR-633-HOXA9 axis *in vivo*.

## Discussion

It has been reported that colorectal adenocarcinoma is the most frequent type of colorectal cancer and is regarded as a serious cause of cancer-related deaths worldwide [20]. To date, more attentions have been focused on this disease due to its heavy burden in human [21]. In this study, we found that PCED1B-AS1 was upregulated in colorectal adenocarcinoma tissues and corresponding cell lines. Moreover, Knockdown of PCED1B-AS1 efficiently inhibited the progression of colorectal adenocarcinoma both *in vitro* and *in vivo*. Our findings revealed that miR-633/HOXA9 mediated the function of PCED1B-AS1 in this cancer.

Previous studies demonstrated that lncRNAs could exert crucial functions by directly sponging miRNAs [22]. To explore the potential mechanism of this lncRNA in this study, we predicted the downstream miRNAs using Starbase and found that there was a putative binding site between PCED1B-AS1 and miR-633. Moreover, the expression of PCED1B-AS1 was negatively correlated with the expression of miR-633 in cancer tissues. Both luciferase reporter assay and RIP assay confirmed the binding relationship between them. In previous studies, PCED1B-AS1 was shown to participate in the progression of different types of cancer by regulating miRNAs. For instance, PCED1B-AS1 promotes the proliferation and restricts the apoptosis of glioma cells via cooperating with miR-194-5p [23]. PCED1B-AS1 regulates the apoptosis and autophagy of macrophage in active tuberculosis by sponging miR-155 [24]. In addition, miR-19b-5p and miR-19b-2-5p might be regulated by PCED1B-AS1 in non-small-cell lung cancer [25]. These reports suggested that one lncRNA could target multiple miRNAs to regulate the development of different diseases. Therefore, PCED1B-AS1 might also participate in the regulation of colorectal adenocarcinoma through modulating other miRNAs except for miR-633, and this hypothesis should be explored in future studies.

MiR-633 has been reported to act as an oncogene in human cancer such as melanoma [26] or as a tumor suppressor in glioblastoma [27]. In the present study, we demonstrated that miR-633 was a tumor suppressor in colorectal adenocarcinoma. Overexpression of miR-633 exhibited similar protective effect to knockdown of PCED1B-AS1, while downregulation of miR-633 significantly elevated the proliferation and inhibited the apoptosis of colorectal adenocarcinoma cells. It has been reported that miRNAs could bind to the 3′-UTR of downstream mRNAs [28,29]. We found that HOXA9 was a target of miR-633 and rescue experiments revealed that HOXA9 mediated the role of PCED1B-AS1 and miR-633 in the progression of colorectal adenocarcinoma. These results were consistent with the previous findings that HOXA9 might participate in the development and function as the potential biomarker for colorectal adenocarcinoma [30]. Our data further confirmed the upregulation of HOXA9 and verified its role in this disease. Moreover, overexpression of HOXA9 obviously attenuated the inhibitory effect of miR-633 mimics in colorectal adenocarcinoma progression. Increasing reports also suggested that one miRNA could target one or more downstream genes, and then regulate the progression of human disease [31]. Hence, more potential downstream genes that are involved in the regulation of miR-633 and PCED1B-AS1 during colorectal adenocarcinoma progression need to be studied. We also constructed the rice model to confirm the protective role of knockdown of PCED1B-AS1 in colorectal adenocarcinoma, as well as the acceleration of miR-633 inhibitor.

## Conclusion

In summary, our study revealed that PCED1B-AS1 promotes the proliferation of colorectal adenocarcinoma through regulating the miR-633/HOXA9 axis, suggesting that PCED1B-AS1 might be considered as a potential therapeutic target for colorectal adenocarcinoma.

## Supplementary Material

Supplemental Material

## Data Availability

The datasets used analyzed during the current study are available from the corresponding author on reasonable request.

## Abbreviations

Long non-coding RNA Lnc RNA

MicroRNA MiRNA

microRNA-633 miR-633

PC‑esterase domain containing 1B‑antisense RNA 1 PCED1B‑AS1

Quantitative reverse transcription PCRs R T-qPCRs

RNA immunoprecipitation RIP

Short hairpin-RNA negative control sh-NC

Short hairpin-RNA PCED1B-AS1 Sh-PCED1B-AS1
