# Supplementary material for: Long non-coding RNA PCED1B-AS1 promotes the proliferation of colorectal adenocarcinoma through regulating the miR-633/HOXA9 axis
Source: Bioengineered. 2022 Feb 17;13(3):5407–20. doi: 10.1080/21655979.2022.2037225 (PMC8974004; doi:10.1080/21655979.2022.2037225)
Supplement: Supplemental Material [file KBIE_A_2037225_SM1854.docx]

**Table 1. The relationships between** **PCED1B-AS1 expression and** **clinicopathological characteristics of** **colorectal adenocarcinoma patients.**

| Characteristics | Expression of PCED1B-AS1 | | P value |
| --- | --- | --- | --- |
|  | Low(n=20) High(n=20) | |  |
| Age |  |  | 0.4652 |
| ≤ 50 | 4 | 6 |  |
| > 50 | 16 | 14 |  |
| Lymph node mestasis |  |  | 0.7357 |
| Yes | 6 | 7 |  |
| No | 14 | 13 |  |
| Pathological Staging |  |  | 0.0565 |
| I + II | 12 | 6 |  |
| III + IV | 8 | 14 |  |
| Distant metastasis |  |  | 0.7491 |
| M0 | 11 | 12 |  |
| M1 | 9 | 8 |  |

Low/high by the sample mean. Pearson χ2 test.
